# Enhancing care coordination for neurofibromatosis type 1 in primary care: insights and applications for rare diseases

**DOI:** 10.1007/s12687-025-00811-5

**Published:** 2025-07-03

**Authors:** William Evans, Jaynee Chauhan, Aliza Imam, Judith Hayward

**Affiliations:** 1 Yorkshire Regional Genetic Service, Chapel Allerton Hospital, Leeds, LS7 4SA UK; 2 NIHR School for Primary Care Research, Centre for Academic Primary Care, University of Nottingham, Nottingham, NG7 2RD UK; 3 Manchester Centre for Genomic Medicine, St Mary’s Hospital, Manchester, M13 9WL UK

**Keywords:** Rare disease, Care co-ordination, Primary care, Neurofibromatosis type 1, Integrated care

## Abstract

Patients with rare diseases often encounter significant challenges, including poor coordination of healthcare services. The UK Rare Disease Framework emphasizes key priorities such as faster diagnoses, greater awareness among healthcare professionals, improved care coordination, and better access to specialist care. This National Health Service (NHS) project, based in the North East and Yorkshire Genomic Medicine Service (GMS), aimed to improve care coordination for patients with rare genetic diseases in primary care. The project focused on developing a generic clinical pathway to improve care coordination and transitions of care that could be applied to a range of rare diseases. Additionally, it sought to strengthen the integration between genomic services and primary care, fostering a more cohesive approach to patient management. The project mapped clinical care pathways for two exemplar rare genetic diseases, Achondroplasia and Neurofibromatosis type 1 (NF1), this paper describes the NF1 pathway and broader learning from this project. The pathways focussed on identifying common clinical touchpoints with primary care and transitions between primary and specialty care. Key findings included the identification of gaps in care coordination, particularly during the transition from paediatric to adult services, and the development of a set of principles and a template for mapping other rare diseases. Feedback from a wide range of stakeholders, including clinicians across specialties and patient representatives, informed the refinement of the pathways. This project illustrates a systematic approach to enhancing care coordination for patients with rare genetic diseases through the mapping of clinical pathways and the development of primary care resources. The principles and template created can be adapted for other rare diseases, enabling the development of concise, disease-specific pathways. By prioritizing care coordination and transitions, and engaging a wide range of stakeholders in the process, this approach offers significant potential to improve the management of rare disease patients, especially during the critical transition from paediatric to adult care.

**Supplementary Information:**

The online version contains supplementary material available at 10.1007/s12687-025-00811-5.

## Highlights

Mapped a primary care focused pathway for Neurofibromatosis type 1.

Identifies crucial integration points for primary care in rare disease management.

Outlines a systematic approach to aid non-specialists in rare disease care.

Develops principles and a template that can be utilized to care pathway map other rare diseases.

**Supplementary Information:**

The online version contains supplementary material available at 10.1007/s12687-025-00811-5.

## Introduction

Rare diseases (RD), defined in Europe as affecting fewer than 5 in 10,000 in the general population (Department of Health and Social Care 2021; Evans and Rafi 2016), are individually rare but collectively common, with a combined point prevalence of 3.5–5.9% (Nguengang Wakap et al. 2019). There are an estimated 3.5 million patients in the UK affected by a RD, with RD recognised as a public health priority both in the UK and globally (Baynam et al. 2024; Department of Health and Social Care 2021; UN 2021). Patients with rare diseases often face common challenges, including delays in receiving an accurate diagnosis, leading to delayed access to treatments and missed opportunities for preconception counselling for the patient and wider family, limited access to clear information about their condition, a lack of therapeutic options, and poor coordination of care. These issues are widely recognized and are reflected in the four priorities outlined in the UK Rare Disease Framework. (Department of Health and Social Care 2021):

- helping patients get a final diagnosis faster.
- increasing awareness of rare diseases among healthcare professionals.
- better coordination of care.
- improving access to specialist care, treatments, and drugs.

Effective care coordination enables the delivery of structured and consistent support for patients, but currently delivery of such care remain inconsistent with many components missed or infrequently implemented (Walton et al. 2020). This leads to patients missing important clinical reviews, the need to navigate multiple specialist clinic appointments across different hospital trusts without centralized oversight, specialists focussing on their field of interest in isolation without thought of the broader context (Baynam et al. 2024), leaving the patient and their families to coordinate care themselves (Rare disease UK 2016). This fragmented approach leads to delays in diagnosis and treatment, redundant tests, conflicting medical advice, and increased travel burdens (Rare disease 2016; Walton et al. 2020).

**Fig. 1:**
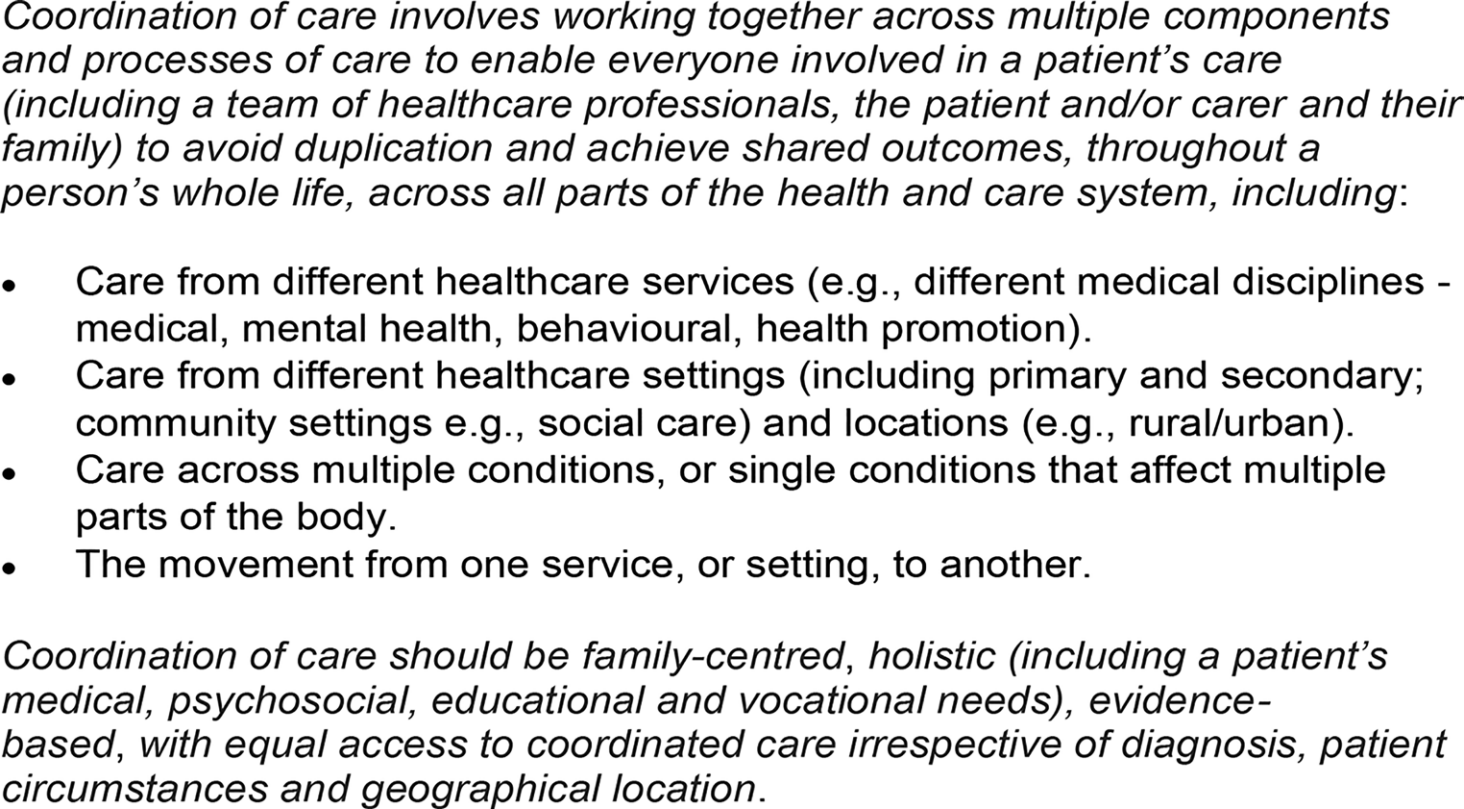
Definition of care coordination (5)

The CONCORD study has defined ‘good’ rare disease care coordination (Walton et al. 2020) (Fig. 1), as well as developing hypothetical models of care coordination (Walton et al. 2022).

International policies and regional initiatives aimed at improving rare disease coordination include the establishment of disease registries, disease networks, specialist centres (Dharssi et al. 2017), and national rare disease care pathway projects (Ward et al. 2022). In England, the 2023 Rare Disease Action Plan outlined several measures to enhance care coordination. These include defining care coordination for all rare disease patients in both new and revised service specifications (not limited to highly specialized services); establishing clear pathways for accessing specialist care, treatments, mental health support, and educational resources; and ensuring the reporting of data to the National Congenital Anomaly and Rare Disease Registration Service (NCARDRS) (Dept of Health and Social Care 2023). The 2024 Action Plan highlights the importance of improving the transition from paediatric to adult care (Dept of Health and Social Care 2024). Previous research on care transitions for rare diseases recommends assigning a designated coordinator to oversee the process before, during, and after the transition, starting the process early, and increasing the use of guidelines and benchmarks. It also emphasizes raising awareness of rare diseases among adult healthcare teams, developing a coordinated transition plan, and implementing annual reviews beginning at age 13 to discuss and refine this plan (CamRare 2022; NICE 2016).

In the UK, the transition of rare disease patients to adult services often involves a shift in care coordination from their paediatrician to their general practitioner (GP). However, this responsibility may remain with specialized care if the disease primarily affects a single organ system, with the relevant specialist overseeing coordination. In cases where dedicated services exist for specific conditions, such as inherited metabolic disorders, care coordination is typically managed within those specialized services. In some locations and situations, a rare disease specialist nurse may assume the role of care coordinator. However, for most patients with rare genetic multi-system diseases, these options are unavailable, leaving care coordination to their general practitioner (GP). Frequently, GPs are unaware of this responsibility, lack the capacity to take it on, or feel unprepared due to insufficient, non-primary-care-focused resources. To bridge this gap, tailored resources designed specifically for primary care clinicians must be developed and made readily accessible. Previous studies indicate that in the context of rare and genomic scenarios, resources for primary care should be concise, relevant, and specifically tailored to primary care needs. They should be integrated into trusted and familiar online repositories (Evans et al. 2020), and prioritize “just-in-time” tools—those highlighting disease “red flags” and designed for immediate use during patient consultations—over “just-in-case” resources intended for broader, less urgent reference (Vandeborne et al. 2019).

In this project a generic clinical pathway was developed that focusses on care co-ordination and care transitions which can be applied to a range of rare diseases, with a focus on the role of primary care. The model was developed through the learning and experience of mapping two exemplar rare genetic diseases, Achondroplasia and Neurofibromatosis type 1 (NF1). The NF1 pathway, and generic pathway are presented here. Mapping an existing healthcare pathway is a well-established method to describe the care of patients, detailing each episode of care, the associated activities, the settings, and the clinicians involved. This approach provides a broader perspective on care delivery, helping to identify gaps, opportunities for improvement, and the development of new services and resources (NICE 2015).

## Methods

### Aims

This UK project, based in the North East and Yorkshire Genomic Medicine Service (GMS), aimed to improve care coordination for patients with specific rare genetic diseases. It sought to establish principles and develop a pathway template to facilitate the efficient mapping of other rare diseases.

The project was devised and led by two practicing general practitioners GPs with additional roles as GPs with a specialist interest in clinical genetics (GPwSI) (WE, JH). Collaborating with the lead clinical geneticists from the region’s three clinical centres, Achondroplasia and Neurofibromatosis type 1 (NF1) were chosen as exemplar diseases. These conditions were chosen for their potential to serve as models, providing a foundation for developing a generalized approach to rare disease pathway mapping.

**Fig. 2:**
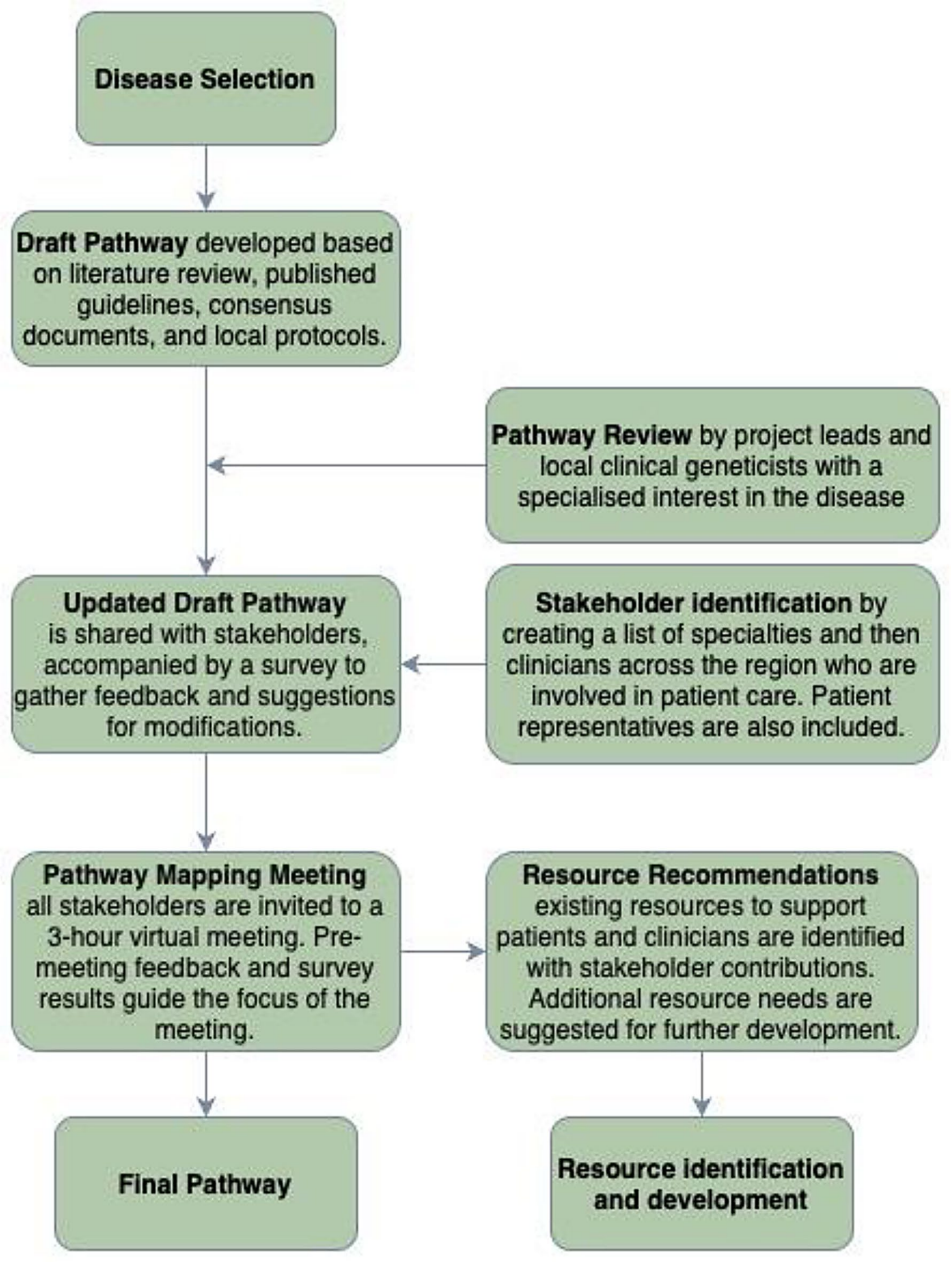
Overview of pathway mapping process for both NF1 and Achondroplasia

The mapping of care pathways for Achondroplasia and NF1 was conducted by two clinical genetic trainee physicians (JC & AI) with familiarity of the diagnosis and management of each disease. A summary of the mapping process is provided in Fig. 2. This paper focuses on the mapping of the NF1 care pathway.

### NF1

The initial draft of the pathway was developed using published literature and existing resources, including materials from highly specialized NF1 services, to capture current good practice in our health setting. This draft was further refined to reflect local practice and resources, with input from primary care clinicians and a clinical geneticist with expertise in NF1.

This draft, accompanied by a link to a questionnaire with some specific questions to steer respondents to areas lacking clarity, was circulated to 23 stakeholders from across the region. This group included representatives of a patient support group, clinical geneticists, paediatricians, general practitioners, neurosurgeons, an ophthalmologist, plastic surgeons, dermatologist, spinal surgeons, neurologists (including from a national specialist centre involved with the care of patients with complex NF1), NF1 clinical nurse specialists, orthopaedic surgeons, and adult and paediatric oncologists.

Feedback was received from 16 stakeholders, with 10 participating in a dedicated workshop (not including the core team) and others providing input outside of the meeting (Table 1). The meeting was held with the core team meeting in person and others joining virtually. The agenda was set as such to enable participants to join the meeting to address questions related to their specialty. The meeting was moderated by the authors. During the meeting the pathway was presented and then reviewed offering opportunity for participants to flag areas of concern, ensuring time was spent on areas previously identified as unclear or ambiguous in the initial pathway development or prior to the meeting in feedback or via the questionnaire.

**Table 1:** Stakeholder involvement in mapping process

	Response to questionnaire (*n*)	Involvement in workshop (*n*)
General practice	2	1
Paediatrics	2	1
Oncology	2	1
Clinical genetics	2	2
Clinical Nurse specialist	3	2
Spinal Surgeon		1
Ophthalmology	1	1
Neurosurgery	1	
Neurology	1	
Patient group representative	1	1

### Template & principles for pathway mapping and development

The insights and experiences gained from this process were distilled into a set of guiding principles and a draft template designed to support the mapping of care pathways for other rare diseases and the development of similar resources. These focus on supporting primary care and community services in managing adolescents and adults with rare diseases, providing practical tools to enhance care delivery and coordination.

## Results

### NF1 pathway

The final draft of the mapped pathways for children and adults with NF1 is included in Appendix A. These pathways were developed using the NF1 review checklist from the Manchester Centre for Genomic Medicine (Manchester Centre for Genomic Medicine 2019) as a foundational framework. By aligning with the layout of this established resource, we aimed to enhance user familiarity and ease of use.

Additional content was incorporated from NF1 care guidelines, consensus documents (Carton et al. 2023; Ferner et al. 2007; Friedman 1993; Miller et al. 2019; Stewart et al. 2018), and local care protocols. The pathway mapping followed an iterative process, as summarized in Fig. 2.

Working drafts were shared with stakeholders, including representatives from a patient advocacy organisation and clinicians managing various aspects of the disease across different clinical settings and localities in the region. The stakeholder meeting specifically addressed unresolved issues and areas lacking consensus on what was best practice in our local health care setting, ensuring a comprehensive and collaborative development process. The final mapped pathway captured current good care in the context of available local resources, existing healthcare structures and referral pathways.

**Fig. 3:**
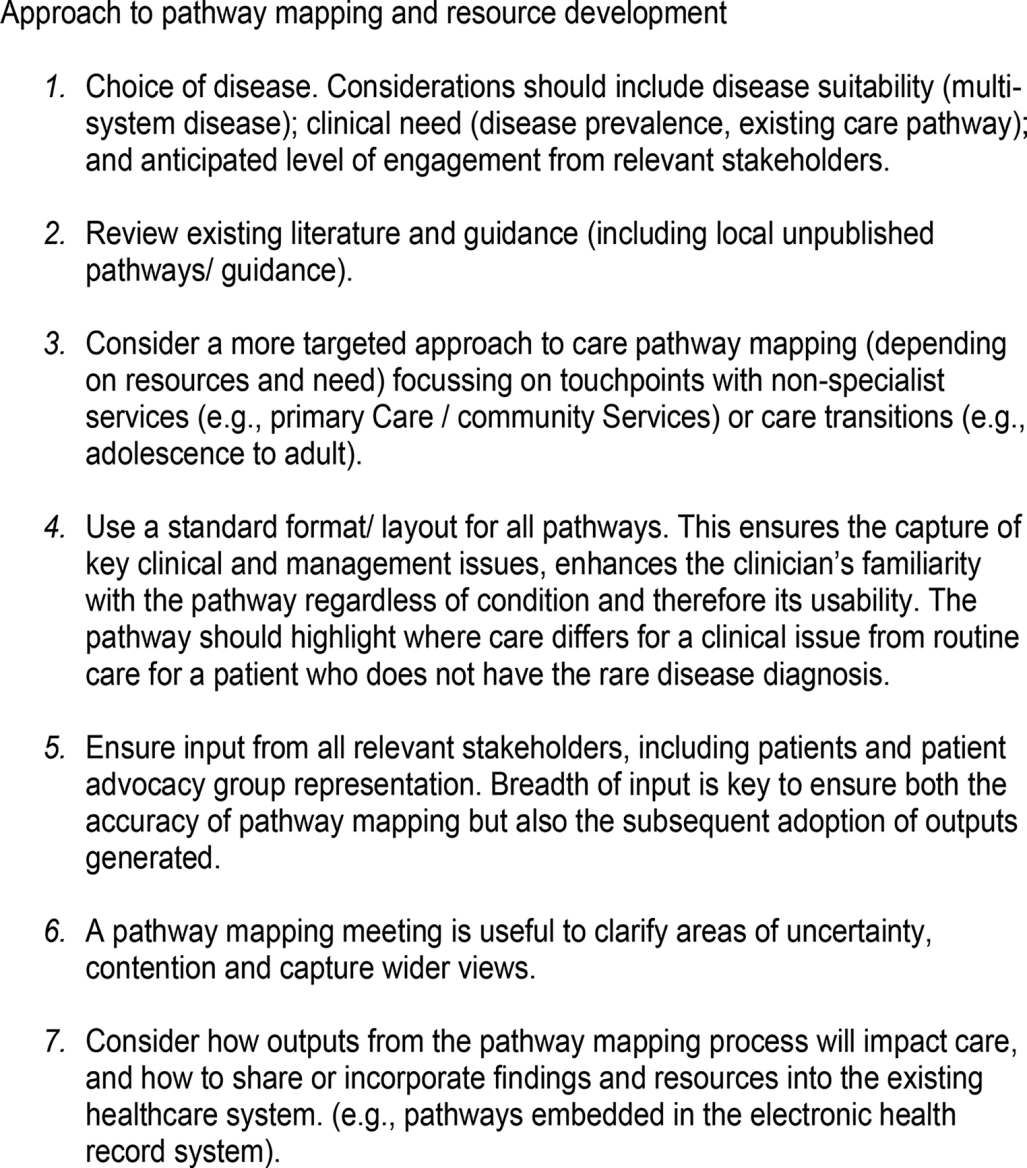
An approach to care pathway mapping

**Fig. 4:**
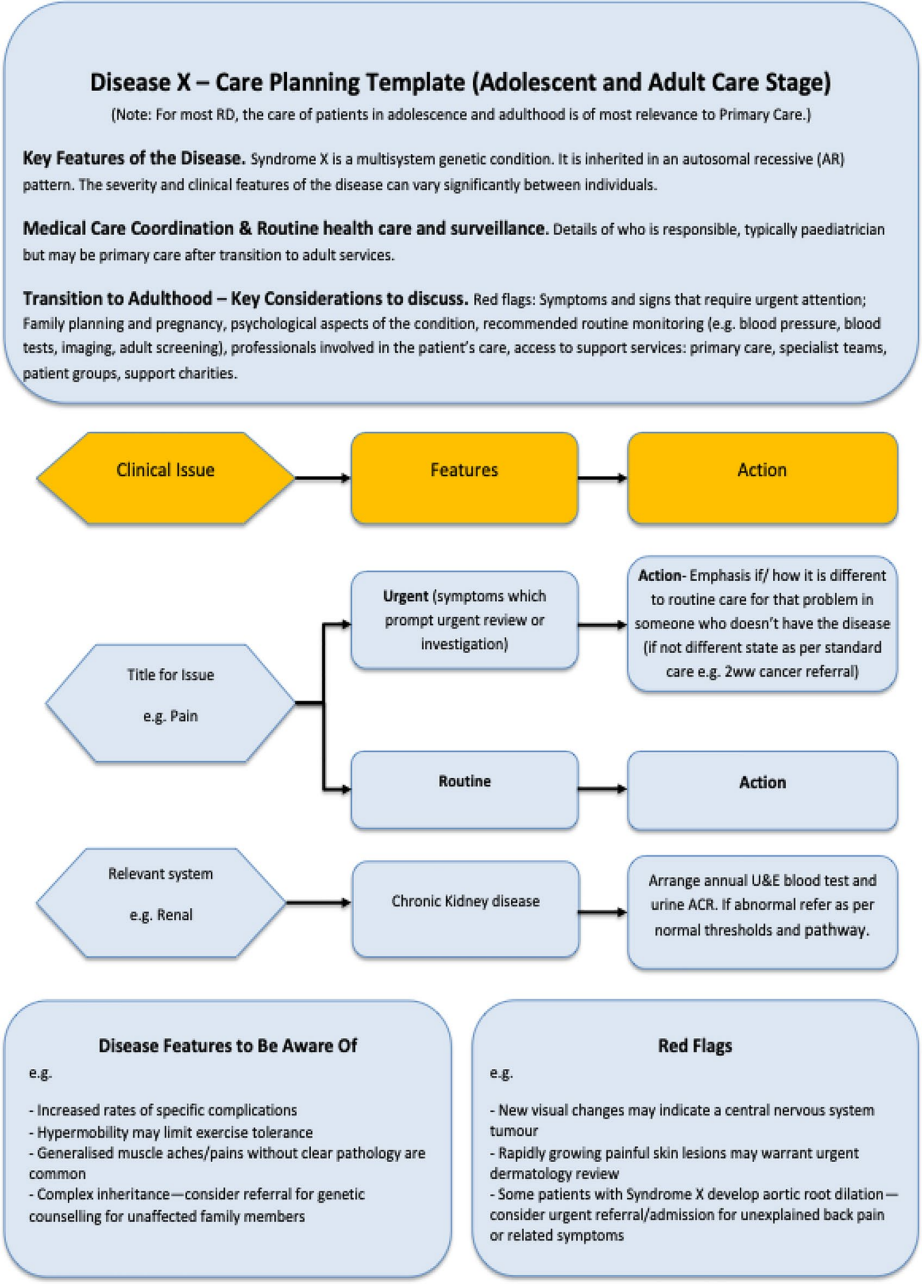
Rare disease pathway mapping template

The principles of approach can be found in Fig. 3 and the generic template in Fig. 4.

## Discussion

This project demonstrates a systematic approach to pathway mapping and resource development aimed at supporting non-specialists in the care of patients with rare diseases. We believe this targeted approach—focused on mapping the pathway and developing resources specifically for primary care clinicians—is unique and distinct from broader initiatives that aim to map the entire healthcare journey (JARDIN WP6 Task 6.2, 2025; Ward et al. 2022).

We identify key challenges encountered during this process and propose strategies to address them. The insights gained are summarized in seven guiding principles for mapping other rare diseases (Fig. 3) and have informed the creation of a generic pathway template (Fig. 4). Although developed for rare diseases, these principles and template could also be applied to other chronic conditions with a need to improve the coordination of care in primary care. However, they were designed with certain considerations that although not unique to rare diseases are a common feature: that they frequently affect children, involve multiple organ systems, and how there are often gaps in knowledge about how to care and manage patients (Walton et al. 2020).

These seven principles align closely with the steps outlined in the recently published care pathway toolkit from the Joint Action on the Integration of European Reference Networks (ERNs) into National Health Systems (JARDIN project) (JARDIN WP6 Task 6.2, 2025). This includes an iterative process for pathway development with steps that include selecting a relevant topic, identifying key stakeholders, incorporating the patient voice into pathway development, clearly defining the scope of the pathway (in this case, a primary care focus), and capturing existing and local clinical practices alongside the published literature.

### Background to 7 principles

1. *Choice of disease. Considerations should include disease suitability (multi-system disease); clinical need (disease prevalence, existing care pathway); and anticipated level of engagement from relevant stakeholders.*

Achondroplasia and NF1 were selected as exemplar diseases due to their multisystem involvement and the need for coordination across multiple clinical specialties. These conditions are considered “relatively common” among rare diseases, with NF1 affecting approximately 1 in 3,000 individuals and Achondroplasia occurring in 1 in 25,000 live births (Orphadata 2023). Both diseases have existing resources, such as guidance documents and expert consensus statements, which provided a strong foundation for the pathway mapping process.

There were some important differences. Mapping the NF1 care pathway was less challenging for several reasons: the availability of guidance, less variation in approach to care across the region, the existence of a nationally commissioned service for complex NF1 patients and regional access to the NF1 nurse specialist service. Even though the NF1 pathway mapping was focused on non-complex NF1 patients, we felt the existence of the nationally commissioned service for complex patients (Nerve Tumours 2023), helped to bring agreement amongst clinicians as they were familiar with defining thresholds for referral and follow up. Additionally, the nurse specialist service across the region has helped to standardise care and approach for patients with NF1. In contrast, Achondroplasia presented greater variation in current practice across the region, reflecting uncertainty among specialists regarding standard routine care and the management of complications. This variability made defining the Achondroplasia care pathway—beyond the scope of this publication—a more complex and challenging process.

2. *Review existing literature and guidance (including local unpublished pathways/ guidance).*

In addition to standard literature search methods, early engagement with key regional experts is crucial to ensure comprehensive information capture. When trying to map what is local good practice, much of the knowledge and information reflecting regional practices including informal guidance is not available through traditional search methods highlighting the importance of expert input in the pathway development process. Although this work did not include a formal appraisal of the literature and guidelines, its primary aim was to document existing practice rather than to develop a new guideline. Future efforts, however, should consider systematically evaluating the quality of the evidence used to inform the pathway, using established tools such as AGREE II (Brouwers et al., 2010; JARDIN WP6 Task 6.2, 2025).

3. *Consider a more targeted approach to care pathway mapping (depending on resources and need) focusing on touchpoints with non-specialist services (e.g., primary care / community services) or care transitions (e.g., transition from paediatric to adult services).*

For both NF1 and Achondroplasia, the complexity of care and the time needed to map the pathways were significantly greater for paediatric populations. This likely reflects several factors, including the breadth and severity of complications that arise during childhood, the more extensive evidence base for paediatric management, and the higher level of engagement by paediatricians in the care of affected children. Paediatricians usually play a pivotal role as care coordinators for these conditions. For instance, children with non-complex NF1 are typically seen annually by paediatric services, often by a paediatrician with a specific interest in NF1 who has developed expertise in the condition’s surveillance and management. After the transition to adult services, significantly less guidance is available for managing conditions like NF1 and Achondroplasia. This gap is further compounded by the shift in care coordination responsibility from paediatricians to primary care providers. This scenario is not unique to NF1 and Achondroplasia but is common across many rare genetic diseases. As the purpose of this pathway mapping process is to provide concise practical information for primary care clinicians, and to ensure the process is as efficient as possible, we recommend prioritizing the mapping of pathways and development of resources for adolescent and adult patients, where the need for structured guidance and support is greatest.

4. *Use a standard format/ layout for all pathways. This ensures the capture of key clinical, and management issues, enhances the clinician’s familiarity with the pathway regardless of condition and therefore its usability. The pathway should highlight where care differs for a clinical issue from routine care for a patient who does not have the rare disease diagnosis.*

The aim of this project was to map exemplar diseases to develop an approach applicable to multiple rare genetic conditions. A simple, standardized, and easy-to-follow format is essential. The layout should highlight common issues, including ‘red flag’ features, and provide clear, actionable guidance. This guidance should emphasize deviations from standard care pathways, specifying unique management requirements or referral protocols (e.g., adults with NF1 and hypertension should be referred for secondary cause investigations).

For non-experts, the resource must be accessible and interpretable during consultations as a ‘just-in-time’ tool (Evans et al. 2020), complementing existing genomic educational resources such as GeNotes (NHS Health Education England 2024). The standardised format enhances usability, fostering familiarity even when the disease-specific content is new.

5. *Ensure input from all relevant stakeholders, including patients and patient advocacy group representation. Breadth of input is key to ensure both the accuracy of pathway mapping but also the subsequent adoption of outputs generated.*

Stakeholder voice from clinical specialities was broad and incorporated the core and many of the additional specialties identified in previous NF1 care pathway work (Ward et al. 2022). We were pleased with the overall level of engagement. However, there was some initial uncertainty about the project’s aims, requiring the team to repeatedly clarify that it focused on pathway mapping rather than creating a new pathway. Additionally, clinicians from one part of the region were underrepresented. Actively recognising and integrating the unique perspectives of patients, parents, and caregivers remains a vital element of this work. The involvement of clinical nurse specialists and a patient group representative in the NF1 pathway was extremely valuable, offering insights across a range of patient experiences. However, direct engagement with patients themselves should be encouraged. This can be supported through the use of tools and resources outlined in the JARDIN care pathway toolkit (JARDIN WP6 Task 6.2, 2025).

6. *A pathway mapping meeting is useful to clarify areas of uncertainty, contention and capture wider views.*

Engagement in the process was challenging, particularly in coordinating a suitable time for multiple clinicians. Several factors improved participation: holding the meeting virtually while the core team met in person, structuring the agenda to allow specific clinicians to join relevant discussions, and circulating the draft pathway in advance to focus the meeting on unresolved issues.

For future projects, sharing draft pathways with core stakeholders in advance may reduce or eliminate the need for additional meetings. However, for more complex or contentious issues, alternative methods—such as the DELPHI process—may be necessary to facilitate consensus-building (JARDIN WP6 Task 6.2, 2025). Notably, in both exemplar diseases, some clinicians participated only during the meeting and did not provide feedback to the circulated draft via email, or by completing the accompanying form with specific questions for comment.

7. *Consider how outputs from the pathway mapping process will impact care, and how to share or incorporate findings and resources into the existing healthcare system. (e.g., pathways embedded in the electronic health record system).*

From the outset, it is important to define why a disease is being mapped, what the outputs will be, and how these will be utilized and implemented. For NF1, the outputs closely follow the proposed template for future disease mapping, incorporating design principles from the Manchester Centre for Genomic Medicine’s NF1 review guidelines (Manchester Centre for Genomic Medicine 2019). These pathways are tailored to support primary care clinicians in managing patients with NF1 across the region. The most effective integration of this “just-in-time” resource would be as part of a suite of templates accessible directly through the electronic health record (EHR) system. Ideally, this guidance should also be accessible through other familiar repositories such as: NICE’s Clinical Knowledge Summaries (for national guidance), local guideline platforms and rare disease charity web-based resources (Evans et al. 2020). Links and hard copies of these pathways can be disseminated at key clinical touchpoints such as diagnosis and transition to adult services.

This project has underscored the critical importance of effectively managing the transition from paediatric to adult services, a period when the quality of care for patients with rare diseases is often at risk of compromise (CamRare 2022; NICE 2016). The NF1 service across the region provides a transition review appointment to identify active clinical issues, arrange appropriate ongoing follow-up, and offer an opportunity to address questions while educating young adults about their condition. These appointments are highly effective and represent a best practice model that should ideally be extended to all patients with rare genetic diseases. The practicalities of conducting these appointments, including who would lead them, remain unclear given the current demands on healthcare services. While certain issues faced by patients with conditions like NF1 are disease-specific, many challenges are common shared across rare genetic diseases. These include the lack of clear management guidelines for adults, the complexity of managing multiple organ system involvement, and addressing reproductive health and family planning concerns.

Supporting patients with a wide range of rare genetic conditions requires consideration of specialized roles, such as General Practitioners with a Special Interest (GPwSI) and rare disease nurse specialists. These roles, built on core competencies applicable to multiple conditions, could act as primary case managers, care coordinator, advocate and care facilitator for RD patients, ensuring that patient health information is both captured and shared between relevant clinical disciplines (Baynam et al. 2024).

Primary care-focused pathways, such as those developed in this project, play a crucial role in managing rare diseases within the community. Clear guidance on referral criteria, the urgency of referrals, and appropriate specialties will facilitate better care and help shift care delivery closer to the patient’s local community—a key objective of the NHS Long Term Plan (NHS England yr).

### Next steps

The NF1 adult pathway is shared with relevant clinicians during the transition process, additionally it is hosted on NF1 charity web pages- a known site for GPs seeking rare disease information (Evans et al. 2020). Additionally, plans are underway to integrate the pathway into repositories accessible via primary care electronic health records (EHRs).

More broadly, the principal focus, in primary care, should be on supporting adolescents and adults with rare diseases. This project demonstrates that this can be most effectively achieved by developing high-level, concise pathways for rare diseases, incorporating elements from the proposed pathway template (Fig. 4).

These pathways should aim to streamline care delivery, ensuring clarity and consistency for primary care providers managing rare diseases. They should address the following key issues:

- **Care Setting and Responsibility**: Clearly define the care setting and the responsible clinician.
- **Transition Review**: Conduct a comprehensive review at the transition to adult care, addressing the care setting, expected routine monitoring, red flag symptoms, recommended actions, reproductive options, psychological implications, and access to support (e.g., specialist nurses, GPs, third-sector organizations, and patient resources).
- **Symptom and Feature Guidance**: Highlight relevant symptoms and clinical features, including specific actions and their urgency (urgent or routine).
- **Special Considerations**: Identify disease features requiring special attention due to higher prevalence or unique management needs compared to the general population.
- **Red Flags**: Clearly outline disease-specific red flags requiring immediate or specialized action.

With over 7,000 rare diseases, determining the focus for further pathway mapping requires strategic prioritization. Pathway development must account for existing pressures on primary care and the perception that such initiatives may shift unfunded responsibilities from secondary to primary care. In the UK, an effective opportunity to enhance rare disease care coordination without increasing primary care workload or creating new services is to focus on rare diseases associated with a learning disability (LD) phenotype.

In the UK, all patients with a learning disability aged 14 and above are entitled to an annual health check with their GP (NHS England 2024).

These health checks involve a review of physical and mental health, prescriptions, and care needs, supported by a maintained LD register to invite eligible patients. While these reviews address shared issues among individuals with LD, syndrome-specific concerns are often overlooked. Current resources and templates used in these health checks are insufficiently equipped with syndrome-specific prompts and guidance.

A simple improvement would be to develop enhanced resources to populate these templates with appropriate prompts and questions tailored to syndrome-specific issues. Expanding mapped pathways to focus on adolescent and adult patients with rare diseases that lead to LD could significantly improve care. These resources could be seamlessly integrated into annual LD health checks, ensuring a targeted, practical, and scalable approach to supporting rare disease patients within primary care.

This pathway mapping process captures current good practice. To ensure the pathway remains relevant a review date should be set to consider changes in local care provision, referral pathways and treatment options. We suggest a standard review every 3 years, this has been chosen as a pragmatic frequency to balance the need to remain up to date in an often rapidly evolving rare disease space, with an awareness of the associated work load pressures and likely resource to do such a review. However, amendments may be made sooner if a significant pathway change is identified.

## Conclusion

In this project, we tackle the challenge of rare disease coordination, a key priority of the UK Rare Disease Framework. We outline an approach to map disease pathways and develop resources for primary care that can be used across a range of rare diseases to support patients and clinicians. The principles and template developed are designed to enhance patient care, promote community-based management, and ensure timely engagement with relevant clinical specialties.

## Electronic supplementary material

Below is the link to the electronic supplementary material.

Supplementary Material 1

Supplementary Material 2

## Data Availability

No datasets were generated or analysed during the current study.
