# Supplementary material for: Enhancing care coordination for neurofibromatosis type 1 in primary care: insights and applications for rare diseases
Source: J Community Genet. 2025 Jul 3;16(6):747–57. doi: 10.1007/s12687-025-00811-5 (PMC12569206; doi:10.1007/s12687-025-00811-5)
Supplement: Supplementary file 1 — Supplementary Material 1 [file 12687_2025_811_MOESM1_ESM.pdf]

Neurofibromatosis type 1 (NF1) - onward care in adults

Depending on complexity of the patient's NF1, their care is transferred to the appropriate service at transition by the paediatrician or transition clinics:

- **Non-complex NF1 - under the care of primary care physicians**
- **Complex NF1 - under the care of NF1 services Complex service**

Every patient should have an annual blood pressure review

CLIINICAL ISSUES	FEATURES	ACTION
Cardiovascular	Hypertension diagnosis and management as per NICE hypertension guidance - confirmed by ambulatory or 24 hour monitoring	Refer all patients with hypertension for investigation of secondary causes (e.g phaeochromocytoma and renal artery stenosis)
Skin	**Urgent** - any lump that's rapidly growing, persistently painful and/or changes in appearance or texture	Urgent 2 week wait referral
Skin	**Routine** - any lumps causing discomfort, cosmetic concerns, irritation, infection or severe psychiatric distress	Routine referral as per local policy for skin lesions
Pain	Pain persisting for >2 weeks, that causes night time waking or affecting activities of daily living e.g. rapidly growing intra-abdominal nerve tumours	Urgent referral (to appropriate specialty depending on site)
Neurology	New or changing neurology - Headaches, changes to vision, balance, personality, weakness or seizures	2 week wait referral +/- direct access CT/MRI
Neurology	Non-motor peripheral neurological symptoms - parathesia or numbness	Referral to neurology (urgency depends on the severity and rate of progression of symptoms)
Eyes	Changes to visual acuity, colour vision, visual fields, proptosis or new squint	Urgent referral to ophthalmology
Eyes		Annual optometry review + onward referral if necessary
Skeletal	Osteoporosis, osteopenia, vitamin D insufficiency/deficiency.	Recommend supplemental vitamin D +/- blood tests as per regional policy and further investigation if indicated
Reproductive issues	Pre pregnancy	Clinical Genetics referral to be sent as early as possible pre conception - for counselling and onward planning
Reproductive issues	Routine antenatal care - monitor BP and neurofibromas for growth	Routine antenatal care
Women	Increased breast cancer risk - as per NICE moderate risk guidance	Screening and management as per the NICE guidance for moderate risk factors for breast cancer

REFERRAL TO THE COMPLEX NF1 TEAM
Criteria for referrals found at: [Neurofibromatosis Type 1 | Manchester Centre for Genomic Medicine (mangen.co.uk)]
If criteria is not met completely the complex NF1 team are available for advice/discussion of cases or imaging at MDT meetings via the above contact details

NHS
North East and Yorkshire
Genomic Medicine Service

NF1 features to be aware of:

- Learning difficulties and behavioural disorders e.g. ADHD and autism - NF1 patients are at higher risk
- Psychological issues
- Fatigue
- Generalised muscle aches and pains
- Scoliosis, congenital bowing or pseudoarthrosis of long bones usually tibia
- Short stature

Red flags

- Vision: changes to visual acuity, visual fields etc - may indicate an optic glioma or CNS tumour

- Central neurology: headaches, seizures, vision disturbance, gait - may indicate a CNS tumours

- Skin: painful, rapidly growing or changing skin lesions - may indicate malignant peripheral nerve sheath tumour

- Cardiovascular: hypertension, episodic headache, sweating, tachycardia - may indicate phaeochromocytoma

- Pain: new onset, severe in nature, waking from sleep, not resolving after 2 weeks - may indicate malignant transformation of internal neurofibroma

Resources:

- Nerve Tumours UK | Home | Nerve Tumours UK
- Neurofibromatosis Type 1 | Manchester Centre for Genomic Medicine (mangen.co.uk)
- Hypertension criteria resource - Overview | Hypertension in adults: diagnosis and management | Guidance | NICE
- NICE breast cancer guidance
