# Supplementary material for: Enhancing care coordination for neurofibromatosis type 1 in primary care: insights and applications for rare diseases
Source: J Community Genet. 2025 Jul 3;16(6):747–57. doi: 10.1007/s12687-025-00811-5 (PMC12569206; doi:10.1007/s12687-025-00811-5)
Supplement: Supplementary file 2 — Supplementary Material 2 [file 12687_2025_811_MOESM2_ESM.pdf]

Neurofibromatosis type 1 (NF1) - onward care in children

For children less than 5 years old with signs or symptoms suggestive of NF1 - carry out genetic testing for NF1
If testing is negative, review at 5 years old - if they do not meet clinical criteria for NF1 discharge
All children with NF1 should have an annual review with a paediatrician
At each appointment check height, weight, OFC and blood pressure

REFERRAL TO THE COMPLEX NF1 TEAM
Criteria for referrals found at: [Neurofibromatosis Type 1 | Manchester Centre for Genomic Medicine (mangen.co.uk)]
If criteria is not met completely the complex NF1 team are available for advice/discussion of cases or imaging at MDT meetings via the above contact details

CLINICAL ISSUES	FEATURES	ACTION
Skin & soft tissue	**Urgent -** Painful plexiform neurofibromas or rapidly changing masses or suspected Malignant peripheral nerve sheath tumour (MPNST)	Urgent referral to paediatric oncology +/- imaging
	**Routine** - Neurofibromas - multiplying or causing discomfort or psychological effects	Refer as per local pathway
Nervous system	Headaches, changes to vision, balance, personality, weakness or seizures	If new neurology or marked changes in symptoms - Urgent referral to neurology or neurosurgery if concerns of CNS tumours +/- imaging; If changes noted to chronic features refer to neurology +/- imaging
	Non-motor peripheral neurological symptoms, parathesia or numbness	If changes noted to chronic features refer to neurology +/- imaging; If new neurology or marked changes in symptoms - Urgent referral to neurology or neurosurgery if concerns of CNS tumours +/- imaging; Discuss with NF1 team e.g. via NF1 nurse specialist for advice
Eyes	Changes to visual acuity, colour vision, visual fields, proptosis or new squint	Urgent referral to ophthalmology
		Annual ophthalmology review until at least 7 years old then as per local recommendations and pathway
Skeletal	Changes to scoliosis	Whole spine x-ray (PA and lateral) + whole MRI scan - refer to spinal surgeons and flag the patient has NF1
	Skeletal issues problems - pseudoarthrosis, tibial bowing	Orthopaedic referral
	Sarcoma	Local imaging +/- paediatric oncology referral
Cardiovascular	Hypertension	Referral to investigate for secondary causes of hypertension as per local pathway
Development	Any delay or difficulties noted in any of areas of development	Early referral/low threshold for specialty services - paediatrics, speech and language therapists, occupational health,, physiotherapists, liaison with school
Behaviour	Difficulties with behaviour - attention, learning or interaction with others	Refer to community team involving other specialties as needed
Pain	Pain persisting for >2 weeks, that causes night time waking or affecting activities of daily living e.g. rapidly growing intra-abdominal nerve tumours	Urgent referral (to appropriate specialty depending on site)

NHS
North East and Yorkshire
Genomic Medicine Service

At transition (16-17 years old) refer back to Clinical Genetics to determine care settings for on-going care.
During transition between children's and adult services, important areas to discuss:

- **Red flag signs and symptoms to be aware of**
- **Family planning and pregnancy**
- **Psychological aspects of NF1**
- **Routine checks - BP and vitamin D**
- **Support - Primary care, NF1 nurse specialist, patient resources (support charities)**

NF1 features to be aware of:

- Learning difficulties and behavioural disorders e.g. ADHD and autism - NF1 patients are at higher risk
- Psychological issues
- Fatigue
- Generalised muscle aches and pains
- Scoliosis, congenital bowing or pseudoarthrosis of long bones usually tibia
- Short stature

Red flags

- Vision: changes to visual acuity, visual fields etc - may indicate an optic glioma or CNS tumour

- Central neurology: headaches, seizures, vision disturbance, gait - may indicate a CNS tumours

- Skin: painful, rapidly growing or changing skin lesions - may indicate malignant peripheral nerve sheath tumour

- Cardiovascular: hypertension, episodic headache, sweating, tachycardia - may indicate phaeochromocytoma

- Pain: new onset, severe in nature, waking from sleep, not resolving after 2 weeks - may indicate malignant transformation of internal neurofibroma

Resources:

- Nerve Tumours UK | Home | Nerve Tumours UK
- Neurofibromatosis Type 1 | Manchester Centre for Genomic Medicine (mangen.co.uk)
- Hypertension criteria resource - Lurbe E, Agabiti-Rosei E, Cruickshank JK, Dominiczak A, Erdine S, Hirth A, Invitti C, Litwin M, Mancia G, Pall D, Rascher W, Redon J, Schaefer F, Seeman T, Sinha M, Stabouli S, Webb NJ, Wühl E, Zanchetti A. 2016 European Society of Hypertension guidelines for the management of high blood pressure in children and adolescents. J Hypertens. 2016 Oct;34(10):1887-920. doi: 10.1097/HJH.0000000000001039. PMID: 27467768.
